# High-throughput sequencing reveals differential regulation of miRNAs in fenoxaprop-*P*-ethyl-resistant *Beckmannia syzigachne*

**DOI:** 10.1038/srep28725

**Published:** 2016-06-29

**Authors:** Lang Pan, Zhaoyun Wang, Jia Cai, Haitao Gao, Hongwei Zhao, Liyao Dong

**Affiliations:** 1 College of Plant Protection, Nanjing Agricultural University, Nanjing 210095, China; 2 Key Laboratory of Integrated Management of Crop Diseases and Pests (Nanjing Agricultural University), Ministry of Education, Nanjing 210095, China

## Abstract

Non-target site resistance (NTSR) to herbicides is an increasing concern for weed control. The majority of previous studies have focused on metabolic resistance mechanisms of NTSR, but no research exists on gene regulation mechanisms behind herbicide resistance, such as microRNA (miRNA). Here, we identified 3 American sloughgrass (*Beckmannia syzigachne* Steud.) populations containing fenoxaprop-*P*-ethyl-resistant plants. We then constructed small RNA libraries and subjected them to deep sequencing and bioinformatics analyses. Forty known and 36 potentially novel, predicted miRNAs were successfully identified. Of these, we identified 3 conserved, predicted candidate NTSR-determinant miRNAs and their potential corresponding target genes, as well as 4 novel potential miRNAs with high count. Target gene prediction and annotation indicated that these 7 differentially expressed miRNAs potentially play a role in regulating specific stress-responsive genes, very likely related to herbicide resistance. Expression profiles were determined with quantitative real-time PCR. The present study is a novel, large-scale characterization of weed miRNAs. The results should further our understanding of miRNA expression profiles associated with herbicide resistance, allowing for the development of more effective weed management strategies.

Weeds are a major threat in agricultural production systems. Herbicide resistance is now widely recognized as the result of adaptive evolution in weed populations to the drastic selection pressures imposed by herbicide applications^1^. As resistant weeds survive herbicides via a variety of mechanisms, studying these mechanisms can provide useful information for weed management. Currently, 2 major types of mechanisms are known to be involved in herbicide resistance^2^,^3^: non-target site resistance (NTSR) and target-site resistance (TSR). The latter involves structural changes to herbicide-binding sites or increased expression of target proteins, whereas the former refers to any mechanism that is not TSR^4^. Compared with NTSR, TSR is easier to study^3^, and as a result, NTSR molecular mechanisms are poorly understood. Existing research on NTSR has mainly focused on mechanisms related to metabolic resistance^5^,^6^. Thus far, no studies exist concerning gene regulation mechanisms that could lead to herbicide resistance, such as microRNAs (miRNAs), although such mechanisms are suspected to play a major role in NTSR^4^.

MicroRNAs are endogenous, small non-coding RNAs (~22 nt) that pair with target mRNAs to negatively modulate their expression^7^. Previous research has demonstrated that miRNAs are involved in plant responses to abiotic and biotic stresses^8^, particularly the latter^9^. Stress-regulated miRNAs have been implicated in responses to drought, cold temperatures, and salinity^10^,^11^,^12^. As herbicides induce powerful abiotic stress, miRNAs may be one of the mechanisms underlying NTSR, in addition to metabolic enzymes^4^. To date, a growing body of evidence supports the regulatory role of miRNAs in plant stress response^13^,^14^, but while miRNAs may play a role in weed stress response, we still do not know how miRNAs in weed species are regulated, and no miRNAs have been identified or characterised that could be linked to herbicide resistance. However, recent rapid advances in high-throughput sequencing have provided an effective way to identify and estimate the expression profiles of miRNAs in herbicide-resistant weeds.

A suitable model for the examination of herbicide resistance is the American sloughgrass *(Beckmannia syzigachne* Steud.). This plant is an annual winter grass widely distributed in the wheat (*Triticum aestivum* L.) and oilseed rape (*Brassica napus* L.) fields of China. Since the 1990s, fenoxaprop-*P*-ethyl has been extensively and continuously used to control crop weeds, including *B. syzigachne*, and thus selecting for the resistance to fenoxaprop-*P*-ethyl in these *B. syzigachne* plants. In recent years, *B. syzigachne* has become a predominant weed in wheat fields rotated with rice (*Oryza sativa* L.), particularly in the middle and lower reaches of the Yangtze River, which are the main grain-producing areas of China^15^. In our previous study, 3 *B. syzigachne* populations containing fenoxaprop-*P*-ethyl-resistant plants were identified. We identified TSR in the plants of these populations, and cross-resistance patterns suggested that NTSR existed simultaneously with TSR in the same plants^16^. NTSR is considered not only a matter of metablism involves metabolic enzymes but also of regulators such like miRNA^4^. Based on well-established role of MiRNAs in plant stress response^4^, we hypothesized that miRNAs are likely involved in fenoxaprop-*P*-ethyl NTSR of *B. syzigachne*.

In this study, small RNA (sRNA) libraries were constructed using *B. syzigachne* populations and subjected to deep sequencing. Our objectives were to: (a) construct a miRNA resource for *B. syzigachne*, (b) identify known predicted candidate NTSR-determinant miRNAs and their corresponding potential target genes, (c) select novel potential miRNAs with high count that are candidate NTSR determinants. The expression profiles of the predicted miRNAs and their predicted targets were determined using quantitative real-time PCR (qRT-PCR) and further validation.

## Results

### mRNA Sequence Data and Assembly

The Illumina sequencing platform was used to sequence the 4 cDNA samples (populations) of *B. syzigachne* and each population generated more than 4 Gb (gigabases) of transcriptome data. More than 80% of the data exhibited an average quality value ≥30, indicating high accuracy. Each population contained approximately 56% GC contents. Whereas the 4 populations differed in the total number and lengths of transcripts and unigenes, the distribution of lengths was consistent. All reads were combined to generate a unique unigene set valid for all 4 populations. Assembled reads from the 4 populations resulted in 46338 unigenes with a total length of 40.83 Mb, a mean length of 873.18 bp and an N50 length of 1463 bp. Among these 46338 unigenes, 30.06% were ≥1000 bp.

### Construction of sRNA libraries using high-throughput sequencing

To identify the candidate NTSR-determinant miRNAs, we used high-throughput sequencing to construct to construct 4 sRNA libraries from 1 population containing herbicide-sensitive plants (the sensitive ‘S’ population; AFCJ-S) and 3 populations containing resistant plants (the resistant ‘R’ populations; JCWL-R, JCJT-R, and JYJD-R). Excluding reads without sRNA sequences, the totals of individual read numbers for each library are listed in Table 1. The sequences in the 4 libraries ranged from 18 to 30 nt in length, with the majority being 18 to 25 nt. We then obtained clean reads after the removal of adaptor sequences, low quality tags, contaminants, and short RNAs <18 nt (i.e., rRNA, snRNA, snoRNA, tRNA, and unannotated reads) (Table 2).

### Identification of known and novel miRNAs

To quantify miRNA expression abundance in the fenoxaprop-*P*-ethyl-resistant *B. syzigachne*, we used BLASTN to compare the sRNA library with known plant miRNAs in miRBase 16.0. After sequence analysis, we identified 40 predicted miRNAs in the 4 sRNA libraries (Table S1). To distinguish novel predicted miRNA sequences, we mapped all unannotated sRNAs onto the *B. syzigachne* transcriptome sequence data. Potential pre-miRNAs were searched and 36 hairpin-like miRNA precursors were predicted from the 4 sRNA libraries (Table S2).

### Prediction and annotation of miRNA putative target genes

**Figure 1:**
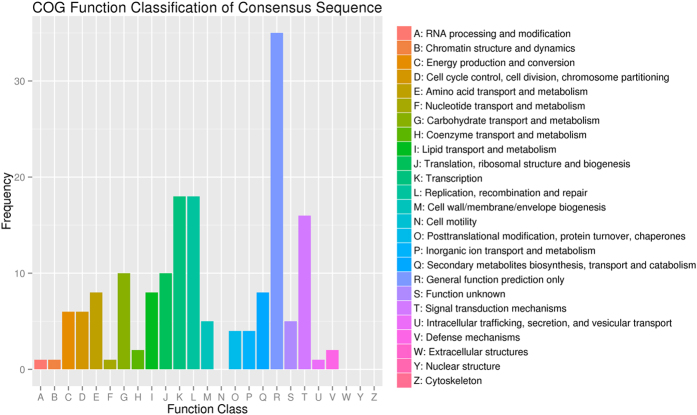
COG classification assigned to miRNA targets in fenoxaprop-*P*-ethyl-resistant *B. syzigachne*. The miRNA target genes were assigned based on the eggNOG database.

We identified and annotated 402 putative miRNA target genes using the transcriptome assembly for *B. syzigachne*. The Clusters of Orthologous Groups (COG) database was used to predict and classify the potential functions of putative miRNA target genes (Fig. 1). All 402 putative target genes were assigned an annotation and 117 of them were functionally classified into 21 COG categories. The cluster for ‘General function prediction only’ was the largest group, followed by ‘Replication, recombination, and repair’ and ‘transcription’.

**Figure 2:**
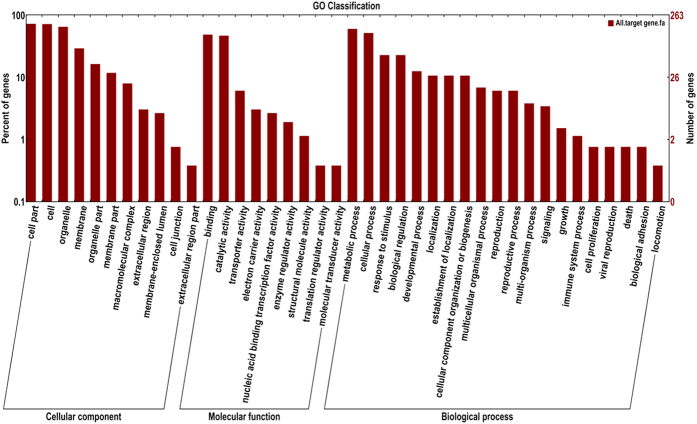
GO classification of miRNA targets in fenoxaprop-*P*-ethyl resistant *B. syzigachne*. The miRNA target genes were assigned using Blast2GO.

To describe the miRNA target gene products in terms of their associated biological processes, cellular components, and molecular functions, we conducted gene ontology (GO) analyses using Blast2GO (Fig. 2). In the biological process, cellular component, and molecular function categories, ‘metabolic process’, ‘cell part’, and ‘binding’ groups were the most highly represented, respectively.

The miRNA target genes were then assigned to unique biochemical pathways using the Kyoto Encyclopedia of Genes and Genomes (KEGG) database. Fifty-five different pathways were found, with ‘metabolic pathways’ being the most represented, followed by ‘biosynthesis of secondary metabolites’.

### Target gene expression analysis of candidate NTSR-determinant miRNAs

We compared the 3 R libraries versus the S library in *B. syzigachne* populations, to identify differences in expression between conserved and novel predicted miRNAs. Based on the criteria described in the ‘Differential miRNA expression analysis’ section of the methods, 7 differentially expressed predicted miRNAs, 3 known and 4 novel, were identified in the 3 R libraries compared with the S library (Table 3). The transcriptome sequences of *B. syzigachne* were used as the reference set to predict the miRNA putative target genes and 7 target genes were identified (Table 3).

### Verification of miRNAs through qRT-PCR

**Figure 3:**
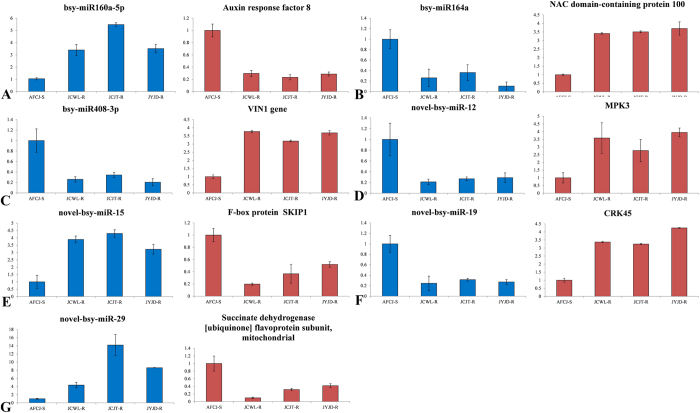
Real-time quantitative PCR analysis of the expression of 7 predicted miRNAs and their predicted target genes. (**A**) bsy-miR160a-5p and its target gene auxin response factor 8 (comp10726). (**B**) bsy-miR164a and its target gene, a NAC domain-containing protein 100 (comp11591). (**C**) bsy-miR408-3p and its target gene VIN1 (comp27035). (**D**) novel-bsy-miR-12 and its target gene MPK3 (comp149885). (**E**) novel-bsy-miR-15 and its target gene F-box protein SKIP1 (comp14355). (**F**) novel-bsy-miR-19 and its target gene CRK45 (comp102833). (**G**) novel-bsy-miR-29 and its target gene succinate dehydrogenase [ubiquinone] flavoprotein subunit, mitochondrial (comp108464).

We performed qRT-PCR analysis on the 7 differentially expressed predicted candidate NTSR-determinant miRNAs and 7 miRNA predicted target genes. Gene expression analysis was performed using 10 pooled RNA samples as described in the methods (‘Plant materials’). The qRT-PCR results indicated that the expression patterns of the predicted miRNAs and their predicted target genes were consistent with the results of Illumina sequencing (Fig. 3, Table 4).

### Further validation of candidate NTSR determinant miRNAs

**Figure 4:**
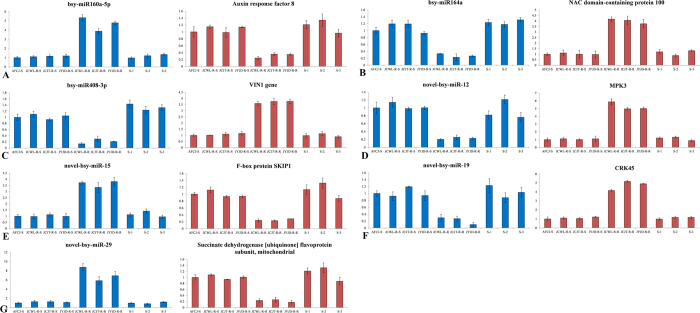
Validation of the expression of 7 predicted miRNAs and their predicted target genes. (**A**) bsy-miR160a-5p and its target gene auxin response factor 8 (comp10726). (**B**) bsy-miR164a and its target gene NAC domain-containing protein 100 (comp11591). (**C**) bsy-miR408-3p and its target gene VIN1 (comp27035). (**D**) novel-bsy-miR-12 and its target gene MPK3 (comp149885). (**E**) novel-bsy-miR-15 and its target gene F-box protein SKIP1 (comp14355). (**F**) novel-bsy-miR-19 and its target gene CRK45 (comp102833). (**G**) novel-bsy-miR-29 and its target gene succinate dehydrogenase [ubiquinone] flavoprotein subunit, mitochondrial (comp108464).

The differences in expression were confirmed in the 7 predicted miRNAs and their predicted target genes were evaluated using individual plants from 10 populations (AFCJ-S, JCWL-R-S, JCJT-R-S, JYJD-R-S, JCWL-R-R, JCJT-R-R, JYJD-R-R, and S1, S2, S3). A susceptibility analysis found that all plants in the JCWL-R-R, JCJT-R-R, and JYJD-R-R lines were resistant, and all plants in the JCWL-R-S, JCJT-R-S, and JYJD-R-S were sensitive. In all resistant lines, TSR and NTSR existed simultaneously and NTSR was never found separately from TSR in any plant. Gene expression analysis was performed using 10 pooled RNA samples as described in the methods (‘Plant materials’). We then used real-time PCR to study the expression of these predicted miRNAs and predicted targets (Fig. 4, Table 5). The results obtained from these additional lines were identical to the results from the JCWL-R, JCJT-R, JYJD-R, and AFCJ-S lines (Figs 3 and 4, Tables 4 and 5).

**Figure 5:**
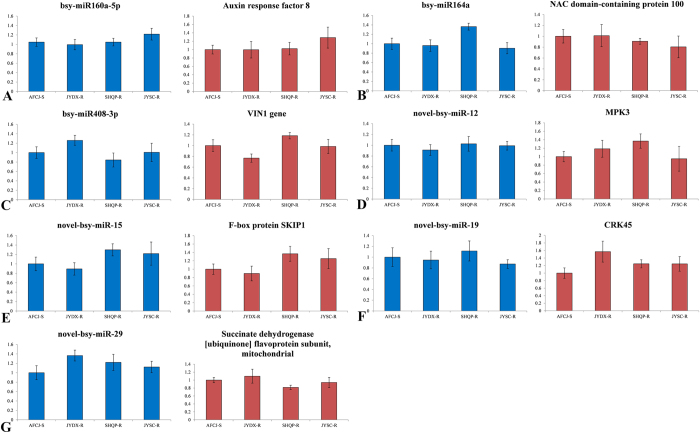
Real-time quantitative PCR analysis of the expression of 7 predicted miRNAs and their predicted target genes for JYDX-R, SHQP-R, JYSC-R and S. (**A**) bsy-miR160a-5p and its target gene auxin response factor 8 (comp10726). (**B**) bsy-miR164a and its target gene NAC domain-containing protein 100 (comp11591). (**C**) bsy-miR408-3p and its target gene VIN1 (comp27035). (**D**) novel-bsy-miR-12 and its target gene MPK3 (comp149885). (**E**) novel-bsy-miR-15 and its target gene F-box protein SKIP1 (comp14355). (**F**) novel-bsy-miR-19 and its target gene CRK45 (comp102833). (**G**) novel-bsy-miR-29 and its target gene succinate dehydrogenase [ubiquinone] flavoprotein subunit, mitochondrial (comp108464).

In JYDX-R, SHQP-R, and JYSC-R plants that exhibited TSR but not PBO-sensitive NTSR mechanisms, we found that the expression patterns (Fig. 5) of the 7 predicted miRNAs and their potential gene targets differed from those shown in Figs 3 and 4 (the JCWL-R, JCJT-R, JYJD-R, and AFCJ-S lines), confirming that the miRNA expression patterns in the JCWL-R, JCJT-R, JYJD-R, and AFCJ-S populations were due at least to the presence of PBO-sensitive NTSR mechanisms.

## Discussion

This study is the first report to examine miRNAs as a gene-regulating mechanism of herbicide resistance, via comparing miRNA expression profiles related to fenoxaprop-*P*-ethyl response, and is unprecedented in weeds. We obtained more than 8 000 000 sequences with lengths of 18–30 nt from 4 miRNA libraries containing 76 miRNAs. Our incomprehensive miRNA resource was likely the cause of the small miRNA count. When the full *B. syzigachne* genome is available, we should be able to identify more *B. syzigachne* miRNAs.

Individual plants often have different genotypes, implying variation in allelic composition and in gene-expression regulation^4^,^17^. This genotypic variability means that differences in resistance and sensitivity to herbicides may be due to a number of factors that are actually irrelevant to NTSR. Thus, dissimilar genetic backgrounds between resistant and sensitive plants interferes with the search for NTSR alleles^4^. In this study, we compared a single susceptible population with 3 fenoxaprop-*P*-ethyl resistant populations that differed in genetic background. To avoid any false-positive alleles, seven populations were used for validating NTSR-related genes (3 R and S sub-populations from a single resistant population sharing similar genetic background, plus a control S population).

Given available data, we were able to use high-throughput sequencing to identify 3 known predicted candidate NTSR determinant miRNAs (bsy-miR160, bsy-miR164, bsy-miR408), as well as their 3 predicted target genes. Specifically, bsy-miR160 appears to regulate one of the auxin response factor (ARF) genes: ARF8, a negative regulator of growth and development in auxin signalling pathways^8^,^18^. Due to this role, ARF8 has been implicated in plant stress response; for example, similar findings of miR160-mediated ARF regulation were also reported in salt-stressed *Paulownia tomentosa*^19^, drought-stressed *Prunus persica*^20^ and cold-stressed trifoliate orange^21^. Thus, via regulation by bsy-miR160, ARF8 is probably also related to fenoxaprop-*P*-ethyl-resistance in *B. syzigachne*. Specifically, ARF8 may modulate the activity or abundance of transcriptional repressors that interact with regulatory elements in the promoters of plant-defence genes. This action regulates the gene expression of enzymes involved in herbicide detoxification. Continued triggering of ARF8 by fenoxaprop-*P*-ethyl treatment may subsequently lead to the fenoxaprop-specific expression of GSTs or other proteins involved in detoxification and defence^22^. This proposed mechanism is similar to research demonstrating that pre-treatment with 2,4-D results in an increased capacity to metabolise the active ingredient diclofop-methyl when the herbicide was subsequently reapplied^23^.

Additionally, miR164, the second known predicted miRNA identified in this study, was previously shown to be involved in stress responses through regulating the cleavage of NAC proteins^24^, which act in various stress-related regulatory networks^25^, including those that enhance drought and salt tolerance in rice and *Arabidopsis thaliana*^26^,^27^. These same mechanisms may also cause NTSR. Specifically, NAC up-regulation decreases the expression of its downstream gene, encoding a glyoxalase I family protein that functions to detoxify aldehydes through the glyoxalase pathway in *A. thaliana*^27^. Glyoxalase enzymes play important roles in the glutathione-based detoxification of methylglyoxal, a by-product of carbohydrate and lipid metabolism^28^. Therefore, fenoxaprop-*P*-ethyl may work by causing the up-regulation of NAC domain-containing protein 100 in susceptible *B. syzigachne*, reducing its downstream gene and thus herbicide detoxification. In fenoxaprop-*P*-ethyl resistant *B. syzigachne*, as in other plants, bsy-miR164 may perform a similar regulatory function on NAC domain-containing protein 100, down-regulating it to allow for increased detoxification.

Finally, the target genes of miR408 are major participants in metabolism and the removal of reactive oxidative species produced under stress^12^,^25^,^29^,^30^. Based on these previous studies, bsy-miR408 likely influences fenoxaprop-*P*-ethyl resistance through the regulation of metabolic genes. For instance, when *Alopecurus myosuroides AmGSTF1* was expressed in *A. thaliana*, *AmGSTF1* exerted a direct metabolic effect that led to the accumulation of protective flavonoids, as well as changes in *A. thaliana* secondary, xenobiotic, and antioxidant metabolism^31^. Moreover, transgenic plants overexpressing *AmGSTF1* acquired multiple herbicide resistance^31^. In this study, down-regulated bsy-miR408 in R populations may positively up-regulate its target gene VIN1 (VERNALIZATION INSENSITIVE 3-LIKE 1). VERNALIZATION INSENSITIVE 3 and VIN1 are key proteins in the vernalisation pathway; VIN1 lowers the flowering threshold to ensure successful reproduction successful in unfavourable conditions^32^. The up-regulation of VIN1 in *B. syzigachne* may play a similar role, ultimately conferring fenoxaprop-*P*-ethyl resistance through, causing the accumulation of antioxidant metabolites and altering the metabolism of resistant plants compared with susceptible plants. Although it is beyond the scope of this study to examine the specific roles of these miRNAs in the implicated pathways, future investigations should clarify such roles.

In addition to the conserved miRNAs and predicted target genes, we found 4 non-conserved miRNAs and their predicted targets, which were candidate NTSR determinants. The serine/threonine-protein kinase MPK3 and the cysteine-rich receptor-like protein kinase CRK45 were, respectively, the predicted target genes of 2 novel miRNAs (novel-bsy-miR12 and novel-bsy-miR12). Both kinases are involved in various abiotic stress response pathways^33^,^34^,^35^. MPK3 is a key regulator of signalling in response to cold, salinity, and wind, whereas CRK45 is implicated in the response to drought. Although stress response pathways do not necessarily overlap, the fact that MPK3, at least, plays a role in several such pathways suggests that the two kinases could also function in herbicide resistance. Their involvement may be related to protection against antioxidant damage, as a major source of toxicity from fenoxaprop-*P*-ethyl is the generation of hydrogen peroxides through its disruption of primary metabolism. This peroxidising effect of fenoxaprop-*P*-ethyl explains why enhanced expression of *Am*GST2 glutathione peroxidase resulted in resistance to the herbicide^36^,^37^. In resistant *B. syzigachne*, antioxidant function from increased MPK3 and CRK45 expression may give plant cells more time to degrade fenoxaprop-*P*-ethyl. Further research is needed to verify this potential role for MPK3 and CRK45.

Next, we found that the predicted target gene of novel-bsy-miR15 was the F-box protein SKIP1, implicated in the cold-stress response of *Poncirus trifoliata*^38^. Assuming some overlap exists between responses to cold stress and to herbicide stress, then the up-regulation of novel-bsy-miR15 observed in *B. syzigachne* indicates that this miRNA is also a likely candidate NTSR determinant. Finally, although we were able to identify the predicted target gene of the fourth miRNA as succinate dehydrogenase [ubiquinone] flavoprotein subunit, mitochondrial, the function of this protein is unknown. Thus, to understand how the 4 novel predicted miRNAs participate in fenoxaprop-*P*-ethyl resistance, future studies should focus on elucidating the function of their predicted target genes.

Existing research has demonstrated miRNAs silence genes by guiding the degradation of target mRNAs or by repressing their post-transcriptional translation^39^,^40^. Here, we found that 4 of the predicted miRNA target genes were up-regulated in resistant *B. syzigachne*, whereas the other 3 were down-regulated. These expression patterns are consistent with the action of miRNAs as repressors. The up- or down-regulation of the predicted target genes appear to reflect phases of herbicide stress response^5^,^6^,^36^,^41^: the initial shock phase when stress-signalling pathways are triggered and the acclimation phase when plant resources are redirected towards defence establishment. Specifically, ARF8 and SKIP1 down-regulation probably occur during the shock phase, as they influence many aspects of plant growth and development. Once the acclimation phase begins, a simultaneous up-regulation of CRK45, MPK3, and NAC domain-containing protein 100, along with other metabolic genes, may be triggered through a regulatory cascade that functions to strengthen metabolism and provide protection against fenoxaprop-*P*-ethyl. Although it is beyond the scope of this study to examine all aspects of pathways involved in NTSR, we note that fenoxaprop-*P*-ethyl resistance is likely the result of complex interactions between the predicted target genes described here and other, currently unidentified genes.

These patterns conform to earlier observations that stress-related up-regulation of miRNAs may down-regulate their target gene mRNAs, while down-regulation of miRNAs may up-regulate their target gene mRNAs. Both mechanisms serve to increase stress tolerance in the plant through either negative or positive regulation. Negative regulation (via up-regulation of miRNAs) involves represses stress-responsive genes and genes related to processes inhibited by stress. Relatedly, positive regulation (via down-regulation of miRNAs) involves the accumulation of target gene mRNAs that are involved in stress response pathways^14^.

The expression of specific miRNAs was strictly modulated in stressed *B. syzigachne* and in turn, it probably influenced the response to herbicides through the regulation of downstream target gene expression. As such, regulating miRNA expression through genetic engineering can considerably enhance or dampen plant tolerance to abiotic and biotic stresses. For instance, constitutive miR319 expression (not found in this study) leads to enhanced salt and drought tolerance in creeping bentgrass^42^ and enhanced cold tolerance in rice^43^. Thus, the 7 differentially expressed predicted miRNAs we identified should prove beneficial in developing methods to counter herbicide-resistant weeds. For example, similar to the successful use of insecticide synergists in combating metabolic resistance in insect pests, chemical synergists can inhibit enzymes responsible for resistance in plants^44^. Moreover, the simultaneous application of herbicides and chemical synergists on resistant weeds can efficiently control their growth and spread, thus delaying the evolution of resistance. These applications will allow for the development of more effective weed management strategies.

Notably, we do not have sufficient data to definitively conclude that the identified miRNAs and their predicted target genes are NTSR determinants. However, we can say that we have identified NTSR-marker miRNAs, which are expressed in association with NTSR but do not necessarily play a direct role^4^. Functional validation to demonstrate that these miRNAs (and their predicted target genes) are truly NTSR determinants is currently underway in our laboratory.

## Methods

### Plant materials

For transcriptome sequencing and sRNA sequencing, we used 3 *B. syzigachne* populations containing fenoxaprop-*P*-ethyl-resistant plants (JCWL-R, JCJT-R, and JYJD-R) and one population containing fenoxaprop-*P*-ethyl-susceptible plants (AFCJ-S). These populations were collected from different sites located more than 118 km apart. The sensitivity of plants in these populations to herbicide was tested following previously described methods^16^. We confirmed NTSR presence in the 3 populations containing resistant plants, although these plants also exhibited TSR^16^. All plants in JCWL-R, JCJT-R and JYJD-R carry ACCase with a mutation, respectively at codon 1781, 2041, and 2096. Most plants (about 95%) are homozygous mutants. Each population only contains a single type of mutant ACCase allele. A comparison of responses to fenoxaprop-*P*-ethyl and fenoxaprop-*P*-ethyl + PBO (piperonyl butoxide) suggested the co-occurrence of TSR and NTSR in many plants from these populations^16^.

We treated 100 randomly selected plants in JCWL-R, JCJT-R, and JYJD-R with CytP450 inhibitor PBO combined with fenoxaprop-*P*-ethyl (recommended dose 62 g a.i. ha^−1^). We treated the plants twice with PBO, in applications of 2100 g a.i. ha^−1^ in 97 L ha^−1^ water each, yielding a total application of 4200 g a.i. ha^−1^ in 194 L ha^−1^ water^16^. The plants were sprayed using a laboratory sprayer equipped with a flat-fan nozzle to deliver 280 L ha^−1^ at 230 kPa. Two weeks later, in JCWL-R, 7 plants died, 19 showed normal growth, and 74 showed limited growth; in JCJT-R, 5 plants died, 17 showed normal growth, and 78 showed limited growth; in JYJD-R, 9 plants died, 9 showed normal growth, and 82 showed limited growth. Dead plants were considered susceptible to herbicide (NTSR was excluded as we found that all resistant plants contain TSR mutation, thus no resistant plants with only NTSR existed). Normal growth was probably due to only TSR or TSR plus NTSR because of other types of metabolic enzymes (PBO had no impact, indicating that PBO-sensitive NTSR mechanisms are not present). Limited growth probably resulted from both TSR and NTSR (PBO had impact, indicating at least the presence of PBO-sensitive NTSR mechanisms). Controls (from all three populations) exposed to fenoxaprop-*P*-ethyl alone at the same rate as fenoxaprop-*P*-ethyl + PBO did not reveal any plants with limited growth. Depending on the population, 74–82% of the plants exhibited limited growth when exposed to fenoxaprop-*P*-ethyl + PBO, indicating the presence of PBO-sensitive NTSR mechanisms. Thus, at least 74% of plants in JCWL-R, 78% of plants in JCJT-R, and 82% of plants in JYJD-R probably had NTSR.

The plants used for miRNA and mRNA sequencing were all ACCase mutants, most of which also contained unknown NTSR mechanisms. We pooled 10 randomly selected plants from each population and used this combined material for construction of the mRNA library and the sRNA library. This design allowed us to investigate the average transcriptomic pattern and transcript levels of 1 susceptible pool and 3 resistant pools, with the latter containing at least 7–8 NTSR-related plants.

Seeds were planted in pots filled with a 2:1 (wt/wt) mixture of sand and soil (pH 5.6, organic matter content 1.4%). All pots were placed in a greenhouse and kept at 20 °C day/15 °C night with a photoperiod of 12 h light/12 h dark. Agronomic practices were performed as needed to maintain vigorous plant growth.

### mRNA library construction and sequencing

Total RNAs were extracted using TRIzol (Invitrogen Inc., California, USA) from the AFCJ-S, JCWL-R, JCJT-R, and JYJD-R populations following manufacturer protocol. Any potential genomic DNA contamination was removed with RNase-free DNase I (Ambion Inc., Texas, USA). The mRNA-seq library was constructed using the Illumina TruSeq RNA Sample Preparation Kit (Illumina Inc., San Diego, CA, USA). Isolation of mRNA, fragment interruption, cDNA synthesis, adapter addition, PCR amplification, and RNASeq were performed by Beijing BioMarker Technologies (Beijing, China)^45^. Poly-A mRNA was isolated using poly-T oligo-attached magnetic beads and fragmented using divalent cations under an elevated temperature. The cleaved RNA fragments were copied into first-strand cDNA using reverse transcriptase and random primers, while the subsequent second-strand cDNA synthesis was performed using DNA Polymerase I and RNase H. A single ‘A’ base was ligated to the short fragments after purification with the MinElute PCR Purification Kit (Qiagen, Dusseldorf, Germany), in preparation for ligation to the sequencing adapters. Fragments (200 ± 25 bp) were separated using agarose gel electrophoresis and selected for PCR amplification as sequencing templates. Reads of 50 bps were paired and oriented for sequencing using the Illumina HiSeq™ 2000 sequencing platform. The dataset has been deposited in the National Center for Biotechnology Information (NCBI; accession number PRJNA290808).

### sRNA library construction and sequencing

The sRNA library was prepared according to the Illumina^®^ TruSeq™ Small RNA Sample Preparation protocol. In brief, after extraction of total RNAs, 3′ and 5′ adaptors were ligated to the RNA. The cDNA was then generated via reverse transcription with SuperScript II ReverseTranscriptase (Invitrogen) and amplified using PCR with DNA polymerase to construct the sRNA library. We sequenced 4 sRNA libraries from the AFCJ-S, JCWL-R, JCJT-R, and JYJD-R *B. syzigachne* populations. The deep-sequencing dataset has been deposited in NCBI (accession number PRJNA290416).

### mRNA sequencing data processing

To obtain high-quality clean read data for de novo assembly, the raw reads were filtered to remove adapter sequences, low-quality reads with ambiguous ‘N’ bases, and reads in which more than 10% of the bases had a Q-value <20. Based on the assumption that small reads might represent sequencing artefacts, sequences smaller than 60 bases were eliminated^46^,^47^,^48^. High-quality, clean reads were assembled into unigenes in Trinity, a program that is efficient in processing full-length transcripts across a broad range of expression levels and sequencing depths^49^. Subsequently, the unigenes were combined to produce the final assembly for annotation.

### sRNA sequencing data processing

Sequencing reads were generated from the 4 constructed sRNA libraries. The raw sequences were subjected to a standard analysis pipeline to process and identify the sequences representing conserved and novel miRNAs^50^. Low-quality sequences (reads with a base quality <20) were removed, and all sequences <18 nt in length were discarded. Sequences from 18 nt to 30 nt were used for downstream analyses. Then we obtained the clean reads, which were annotated as tRNAs, rRNAs, snoRNAs, miRNAs, intro, and other unannotated reads according to the Rfam (11.0 release) database.

Unique sequences were used in a BLASTN search against the miRNA database (miRBase16.0), and matched sequences (with no more than 2 mismatches) were considered as conserved miRNAs. Because limited transcriptome data exist on closely related weed species, we generated a single unigene assembly of *B. syzigachne* including all transcriptome data as reference. The miRNA nomenclature was added as previously described^13^,^51^. We looked for novel miRNAs (miRNA that is unique to *B. syzigachne* and thus differs from those found in other species) by mapping unannotated unique sequences to the *B. syzigachne* transcriptome data using MIREAP software (http://sourceforge.net/projects/mireap/). The MIREAP parameters were as follows: (1) a characteristic stem-loop structure; (2) the maximal free energy allowed for the miRNA precursor was −20 kcal mol^−1^; (3) the minimum number of common base pairs between miRNA and miRNA* was 16, with no more than 4 bulges; (4) the maximum asymmetry of the miRNA/miRNA* duplex was 4 bases.

### Prediction of potential miRNA target genes and functional annotation

The miRNA putative targets were identified using the transcriptome assembly for *B. syzigachne*. Four miRNA libraries (1 per population) were separately mapped to the transcriptome assembly to predict potential target genes. The rules used for target prediction were based on previous reports^52^,^53^. These rules were: (1) no more than 4 mismatches; (2) no more than 2 adjacent mismatches in miRNA/target duplex; (3) no more than 1 mismatch at positions 1–9; (4) no mismatches at positions 10–11, (5) no more than 2.5 mismatches in positions 1–12 of the miRNA/target duplex (5′ end of miRNA).

Detailed functional information is necessary for elucidating overall expression profiles of potential target genes. Numerous target sequences were assigned to various non-redundant protein (Nr), Swiss-Prot, GO, COG, and KEGG classifications^54^,^55^,^56^,^57^. A BLASTX search was performed against the NCBI Nr database and the SwissProt database with an E-value ≤10^−5^. Gene names were assigned based on the best BLAST hit (highest score), excluding any uninformative descriptions. A BLASTN search was also performed against the NCBI Nt database, GenBank, and RefSeq using a protein query. Next, Blast2GO was used to annotate the assembled sequences and assign GO terms (molecular functions, biological processes, and cellular components) with an E-value ≤10^−5 ^58. The unigene sequences were also aligned to the COG protein database to predict and classify their functions. The KEGG database was used to assign the assembled sequences from KEGG web server (http://www.genome.jp/kegg)^57^. Transcript levels were quantified via counting reads per kb of exon model per million mapped reads (RPKM)^59^, reflecting the molar concentration of a transcript normalized for RNA length and the total number of reads.

### Differential miRNA expression analysis

We compared miRNA expression across the 4 populations to identify differences. We first normalized miRNA expression in the 4 samples (10 plants per population) to obtain the expression of transcript per million reads (TPM). The normalized expression was equal to the actual miRNA count divided by the total count of clean reads, and then multiplied by 10^6^. If the miRNA gene expression was 0 after normalization in any 1 of 2 samples, it was revised to 0.01. Additionally, if the miRNA gene expression in the 2 samples was <1, the sample was not used in the analysis of differential expression to avoid bias from low expression levels. The fold change between 2 samples was calculated as: fold change = log_2_ (sample 1/sample 2)^60^. Two threshold values, t-test (P < 0.05), and 2-fold change in the expression ratios between resistant pools and the sensitive pool were used as the criteria to identify predicted miRNAs potentially linked to NTSR.

### Verification of miRNAs with qRT-PCR

To verify the high-throughput sequencing results in which we identified candidate NTSR determinant miRNAs, 7 differentially expressed miRNAs were selected and analysed with qRT-PCR. Here, we used the same RNA samples that were used for RNASeq. Real-time PCR was performed using the ABI-7500 Fast Real-Time PCR System (ABI, USA) with the SYBR^®^ *Premix Ex Taq*^TM^ kit (TaKaRa, Japan). The relative expression of genes to the control was calculated using the 2^−ΔΔCT^ method^61^. Each experiment included 3 biological replicates and was repeated at least twice. Significant differences in expression levels were analysed using Welch’s *t*-test^62^. Two threshold values, a significant result in the *t*-test (*P* < 0.05) and a 2-fold change, were used to determine either up- or down-regulation. Capsine phosphatase (CAP), glyceraldehyde-3-phosphate dehydrogenase (GAPDH), and ubiquitin (UBQ) were used as internal control genes. The sequences of the putative target genes used for qPCR primer design were determined from the transcriptome assembly of *B. syzigachne*. The primers used for qRT-PCR are listed in Tables 6 and 7. The reverse primer used for miRNA detection was as follows: 5′-GTGCAGGGTCCGAGGT-3′.

### Further validation of miRNAs that were candidate NTSR determinants

Fenoxaprop-*P*-ethyl-resistant and -susceptible lines were separated from the JCWL-R, JCJT-R, and JYJD-R populations to identify 3 pairs of resistant/susceptible lines with similar genetic backgrounds. A total of 300 plants from each population were kept in a greenhouse under conditions of 20 °C day/15 °C night and a photoperiod of 12 h light/12 h dark, and cultivated to tillering in 3-inch pots.

We collected 2 tillers from each individual plant: one was used for susceptibility analysis and the other for seed collection. Susceptibility analysis was conducted using fenoxaprop-*P*-ethyl at the recommended field dose, and survival was assessed 3 weeks after treatment. The surviving tillers were classified as resistant and the dead tillers as susceptible. The tillers used for seed collection were self-pollinated to produce progeny populations. As a result, 15 susceptible and 285 resistant individuals were identified in the JCWL-R population; 15 susceptible and 285 resistant individuals in the JCJT-R population; and 16 susceptible and 284 resistant individuals in the JYJD-R population. All resistant plants were detected with dCAPS method to confirm that they all hold TSR mutation (all resistant plants in JCWL-R population contain Ile-1781-Leu mutation, all resistant plants in JCJT-R population contain Ile-2041-Asn mutation, all resistant plants in JYJD-R population contain Gly-2096-Ala mutation)^16^. We put the confirmed resistant tillers in a pollen mask and the confirmed susceptible tillers in another pollen mask to obtain progenies. Using another susceptibility analysis with the same herbicide dosage, all plants assayed for herbicide sensitivity in 3 susceptible lines (JCWL-R-S, JCJT-R-S, and JYJD-R-S) were susceptible and all plants assayed for herbicide sensitivity in 3 resistant lines (JCWL-R-R, JCJT-R-R, and JYJD-R-R) were resistant. We detected TSR via ACCase sequencing and NTSR via the use of PBO, a P450 inhibitor. Primers used for sequencing were previously reported^16^. The results confirmed that TSR and NTSR co-existed in every resistant line. ‘R lines’ were derived from 284–285 resistant plants while ‘S lines’ were derived from 15–16 plants.

The standard susceptible population (S) was used as controls for the susceptibility analysis. Based on the results of these susceptibility analyses, we added 3 more susceptible populations (S1, S2, S3) to improve the reliability of miRNA identification. The 7 differentially expressed predicted miRNAs were validated and their predicted target genes were evaluated using individual plants from 10 populations, including AFCJ-S, JCWL-R-S, JCJT-R-S, JYJD-R-S, JCWL-R-R, JCJT-R-R, JYJD-R-R, and S1, S2, S3.

To confirm that the expression patterns of the miRNAs were at least the result of PBO-sensitive NTSR mechanisms, we also added 3 populations containing resistant plants that exhibited TSR and lacked PBO-sensitive NTSR mechanisms. These populations were JYDX-R (collected from Daxingcun in Jiangsu Province, 8 years of fenoxaprop-*P*-ethyl use), SHQP-R (collected from Qingpu in Shanghai, 11 years of fenoxaprop-*P*-ethyl use), JYSC-R (collected from Shangcicun in Jiangsu Province, 10 years of fenoxaprop-*P*-ethyl use). Again, we used ACCase sequencing to confirm TSR: all plants in JYDX-R contained the Ile-1781-Leu mutation, all plants in SHQP-R contained the Ile-2041-Asn mutation, and all plants in JYSC-R contained the Gly-2096-Ala mutation. Despite containing very similar frequencies of the same ACCase mutations, these 3 populations were collected from different places with populations JCWL-R, JCJT-R, and JYJD-R.

We treated 50 randomly selected plants from JYDX-R, SHQP-R, and JYSC-R with fenoxaprop-*P*-ethyl + PBO and 20 randomly selected plants with fenoxaprop-*P*-ethyl alone. Fenoxaprop-*P*-ethyl and PBO were sprayed as described under ‘Plant materials’. No phenotypic differences were observed between plants subjected to fenoxaprop-*P*-ethyl alone and fenoxaprop-*P*-ethyl + PBO. Thus, we confirmed that PBO-sensitive NTSR mechanisms did not occur in these plants. We validated the 7 differentially expressed predicted miRNAs and evaluated their predicted target genes using plants from these 3 populations.

Gene expression analysis was performed using 10 pooled RNA samples as previously described (under ‘Plant materials’). The sequences of putative target genes used for qPCR primer design were again determined from the transcriptome assembly in *B. syzigachne*. Real-time PCR was performed as described in ‘Verification of miRNAs with qRT-PCR’.

## Additional Information

**How to cite this article**: Pan, L. *et al*. High-throughput sequencing reveals differential regulation of miRNAs in fenoxaprop-*P*-ethyl-resistant *Beckmannia syzigachne*. *Sci. Rep.* **6**, 28725; doi: 10.1038/srep28725 (2016).

## Supplementary Material

Supplementary Information

## Figures and Tables

**Table 1:** Summary of data produced by small RNA sequencing after initial cleaning.

Sample	Total reads	Low quality	‘N’ reads	Length <18 nt	Length >30 nt	Clean Reads
AFCJ-S	23 102 322	0	1 607	10 578 163	2 891 855	9 630 697
JCWL-R	24 412 341	0	2 162	9 905 970	3 669 050	10 835 159
JCJT-R	21 776 191	0	194	9 886 698	1 590 788	10 298 511
JYJD-R	22 652472	0	1 423	7 000 606	3 436 202	12 214 241

**Table 2:** Distribution of reads across RNA categories in *Beckmannia syzigachne.*

Category	AFCJ-S		JCWL-R		JCJT-R		JYJD-R	
	Number	Percent	Number	Percent	Number	Percent	Number	Percent
Total	9 630 697	100%	10 835 159	100%	10 298 511	100%	12 214 241	100%
miRNA	31 101	0.32%	36 595	0.34%	103 813	1.01%	37 788	0.31%
rRNA	6 196 149	64.34%	7 065 510	65.21%	6 026 448	58.52%	79 96 614	65.47%
snRNA	5 447	0.06%	5 857	0.05%	2 542	0.02%	4 799	0.04%
snoRNA	98	0.00%	68	0.00%	48	0.00%	69	0.00%
tRNA	302 771	3.14%	334 956	3.09%	331 495	3.22%	455 660	3.73%
Unannotated	3 095 131	32.14%	3 392 173	31.31%	3834165	37.23%	3 719 311	30.45%

**Table 3:** Targets and putative functions of differential predicted miRNAs.

miRNA Family	Target gene	Target gene function
bsy-miR160a-5p	comp10726	Auxin response factor 8
bsy-miR164a	comp11591	NAC domain-containing protein 100
bsy-miR408-3p	comp27035	VIN1 gene
novel-bsy-miR-12	comp149885	MPK3
novel-bsy-miR-15	comp14355	F-box protein SKIP1
novel-bsy-miR-19	comp102833	CRK45
novel-bsy-miR-29	comp108464	Succinate dehydrogenase [ubiquinone] flavoprotein subunit, mitochondrial

**Table 4:** The expression patterns of 7 predicted miRNAs and their predicted target genes identified with qRT-PCR (2^−ΔΔCT^) and Illumina sequencing (RPKM/TPM).

	JCWL-R/AFCJ-S		JCJT-R/AFCJ-S		JYJD-R/AFCJ-S	
	RPKM/TPM	2^−ΔΔCT^	RPKM/TPM	2^−ΔΔCT^	RPKM/TPM	2^−ΔΔCT^
bsy-miR160a-5p	4.74*	3.40*	4.98*	5.48*	4.91*	3.52*
Auxin response factor 8	−3.88*^a^	−3.38*	−4.39*	−4.29*	−4.46*	−3.48*
bsy-miR164a	−9.33*	−3.83*	−6.54*	−2.75*	−4.50*	−9.62*
NAC domain-containing protein 100	2.92*	3.41*	3.26*	3.51*	3.25*	3.71*
bsy-miR408-3p	−10.66*	−3.85*	−11.28*	−2.92*	−11.05*	−4.88*
VIN1 gene	3.92*	3.77*	4.05*	3.18*	3.63*	3.69*
novel-bsy-miR-12	−3.63*	−4.74*	−3.61*	−3.73*	−3.58*	−3.45*
MPK3	7.00*	3.59*	8.94*	2.77*	9.41*	3.95*
novel-bsy-miR-15	3.24*	3.89*	3.15*	4.30*	4.01*	3.22*
F-box protein SKIP1	−3.77*	−5.08*	−5.01*	−2.72*	−3.11*	−2.11*
novel-bsy-miR-19	−4.43*	−4.05*	−7.39*	−3.17*	−4.07*	−3.68*
CRK45	4.42*	3.37*	4.03*	3.25*	3.78*	4.24*
novel-bsy-miR-29	5.33*	4.36*	5.60*	14.19*	2.57*	8.62*
Succinate dehydrogenase [ubiquinone] flavoprotein subunit, mitochondrial	−4.08*	−10.10*	−2.45*	−3.15*	−6.13*	−2.40*

*Significant difference (P < 0.05) from GenePattern analysis. ^a^“−” indicates that genes in the R lines were down-regulated compared with genes in the S lines.

**Table 5:** Further validation of candidate NTSR determinant miRNAs using qRT-PCR (2^−ΔΔCT^).

	AFCJ-S	JCWL-R-S	JCJT-R-S	JYJD-R-S	JCWL-R-R	JCJT-R-R	JYJD-R-R	S1	S2	S3
bsy-miR160a-5p	1	1.11	1.17	1.19	5.33*	3.87*	4.78*	0.98	1.21	1.35
Auxin response factor 8	1	1.15	0.98	1.14	0.25*	0.36*	0.35*	1.21	1.34	0.97
bsy-miR164a	1	1.20	1.19	0.92	0.33*	0.23*	0.27*	1.23	1.18	1.31
NAC domain-containing protein 100	1	1.11	1.00	0.97	4.18*	4.06*	3.75*	1.21	0.88	1.32
bsy-miR408-3p	1	1.10	0.93	1.05	0.14*	0.29*	0.21*	1.43	1.23	1.31
VIN1 gene	1	1.01	1.10	1.16	3.60*	3.76*	3.76*	0.98	1.13	0.89
novel-bsy-miR-12	1	1.14	0.98	1.00	0.20*	0.26*	0.23*	0.82	1.21	0.76
MPK3	1	1.12	1.02	1.10	5.87*	4.98*	5.02*	1.21	1.32	0.88
novel-bsy-miR-15	1	0.97	1.12	0.99	3.73*	3.34*	3.82*	1.12	1.41	0.95
F-box protein SKIP1	1	1.12	0.93	0.94	0.24*	0.23*	0.29*	1.13	1.32	0.88
novel-bsy-miR-19	1	0.92	1.20	0.94	0.30*	0.27*	0.10*	1.23	0.88	1.03
CRK45	1	1.10	1.05	1.22	4.17*	5.17*	4.90*	0.99	1.17	1.17
novel-bsy-miR-29	1	1.28	1.26	1.12	8.76*	5.84*	6.91*	0.98	0.82	1.21
Succinate dehydrogenase [ubiquinone] flavoprotein subunit, mitochondrial	1	1.09	0.93	1.01	0.24*	0.26*	0.18*	1.21	1.32	0.88

*Significant difference (P < 0.05) from GenePattern analysis.

**Table 6:** Differential miRNA primers used for qRT-PCR analysis.

miRNA	RT primer (5′–3′)	Forward primer (5′–3′)
bsy-miR160a-5p	GTCGTATCCAGTGCAGGGTCCGAGGTATTCGCACTGGATACGATGGCAT	TTATTGCCTGGCTCCCTGC
bsy-miR164a	GTCGTATCCAGTGCAGGGTCCGAGGTATTCGCACTGGATACGAACACGT	CTGCATGGAGAAGCAGGTC
bsy-miR408-3p	GTCGTATCCAGTGCAGGGTCCGAGGTATTCGCACTGGATACGACTCAGGG	GGTTCGTGCACTGCCTCTT
novel-bsy-miR-12	GTCGTATCCAGTGCAGGGTCCGAGGTATTCGCACTGGATACGACCTACCT	GGCGCCAGAATTATGGAACGT
novel-bsy-miR-15	GTCGTATCCAGTGCAGGGTCCGAGGTATTCGCACTGGATACGACCACGTT	GCGCCGATGGTGTATTACACA
novel-bsy-miR-19	GTCGTATCCAGTGCAGGGTCCGAGGTATTCGCACTGGATACGACGACACT	GGCGCCTCCGATCCAAAATA
novel-bsy-miR-29	GTCGTATCCAGTGCAGGGTCCGAGGTATTCGCACTGGATACGACGGAGCG	ATAATATCGTCTCCGCCGCGT

**Table 7:** Primers for corresponding potential target genes used in qRT-PCR analysis.

Gene	Direction	Sequence (5′–3′)	Amplicon size (bp)
bsy10726	Forward	AACTGAGGAGGCCTGTGCTA	170
	Reverse	ATAAACGGTGCGGTCAGTTC	
bsy11591	Forward	CGTCGTCAGCAGCAAATAAA	234
	Reverse	AGGAGTGATTTTCCGTGGTG	
bsy27035	Forward	ACACGAGCAAGAGGGAAGAA	193
	Reverse	CCCCCAATTTTCACATCATC	
bsy149885	Forward	GGTGATGCTGTCACACGGTA	204
	Reverse	GCACACAGGTGCAGCTTTTA	
bsy14355	Forward	GCGGGCTATTATTTGCGTTA	162
	Reverse	AGCATGTGATCTTGCGTCAG	
bsy102833	Forward	AAGTCCCAAAAAGGGATGCT	177
	Reverse	AAGATCACCTGGTCGGTTTG	
bsy108464	Forward	AGCTCTTCCTCGGTTTCGAT	178
	Reverse	GCAATACTGCAGGGACGACT	
