# Supplementary material for: High-throughput sequencing reveals differential regulation of miRNAs in fenoxaprop-P-ethyl-resistant Beckmannia syzigachne
Source: Sci Rep. 2016 Jun 29;6:28725. doi: 10.1038/srep28725 (PMC4926119; doi:10.1038/srep28725)
Supplement: Supplementary Information [file srep28725-s1.pdf]

Supplementary Information

High-throughput sequencing reveals differential regulation of miRNAs in fenoxaprop-P-ethyl-resistant *Beckmannia syzigachne*

Lang Pan[a,b], Zhaoyun Wang[a,b], Jia Cai[a,b], Haitao Gao[a,b], Hongwei Zhao[a,b], Liyao Dong[a,b,]*

a. College of Plant Protection, Nanjing Agricultural University, Nanjing 210095, China

b. Key Laboratory of Integrated Management of Crop Diseases and Pests (Nanjing Agricultural University), Ministry of Education, Nanjing 210095, China

* *Correspondence to: Liyao Dong, College of Plant Protection, Nanjing Agricultural University, Nanjing 210095, China. E-mail: dly@njau.edu.cn*

Supplementary Contents:

Table S1 Summary of conserved miRNAs

miRNA Family	Name	miRNA Sequence (5' to 3')	Length	Homology by specie
miR156	bsy-miR156a-5p	ugacagaagagagagagcac	20	*A. thaliana*
miR6475	bsy-miR6475	ucuugagagaccaaaaucugc	21	*P. trichocarpa*
miR396	bsy-miR396e-5p	uccacaggcuuucuugaacgg	21	*O. sativa*
miR165	bsy-miR165a-3p	ucggaccaggcuucauucccc	21	*A. thaliana*
miR2111	bsy-miR2111a-5p	aaaucugccaguuuuggcacc	21	*A. thaliana*
miR160	bsy-miR160a-5p	ugccuggcucccugcaugcca	21	*A. thaliana*
miR6032	bsy-miR6032-3p	acugcuggcgaacagaaaaacgug	24	*B. rapa*
miR5022	bsy-miR5022	uucaugggagauguagcuc	18	*A. thaliana*
miR164	bsy-miR164a	uggagaagcaggucacgugu	20	*A. thaliana*
miR1130	bsy-miR1130b-3p	ucuuauaucaggggacagagg	21	*T. aestivum*
miR5281	bsy-miR5281b	ucuuauauuguggaacggagg	21	*B. distachyon*
miR5272	bsy-miR5272a	uaauugaugcagauuuagua	20	*M. truncatula*
miR5080	bsy-miR5080	aaaaggauguaucuagacgu	20	*O. sativa*
miR408	bsy-miR408-3p	ugcacugccucuucccuga	19	*A. thaliana*
miR5174	bsy-miR5174a	cuccguuccauaauauaagcc	21	*B. distachyon*
miR907	bsy-miR907	gcuucugcagguggcuuuuc	20	*C. reinhardtii*
miR5181	bsy-miR5181d	acuuauuauggauuggaggua	21	*B. distachyon*
miR7714	bsy-miR7714-3p	uuaauauggaucggagggagu	21	*B. distachyon*
miR167	bsy-miR167c	ucagaugacugcucuuuc	18	*G. raimondii*
miR827	bsy-miR827-5p	auuuguuggagaugguguuga	21	*B. distachyon*
miR2907	bsy-miR2907a	cgcagccgucgccugugggagcgg	24	*O. sativa*
miR536	bsy-miR536a	uucgugccggcaaugaacgcguc	23	*P. patens*
miR7776	bsy-miR7776-5p.1	uuugcccucccucucuaucua	21	*B. distachyon*
miR5387	bsy-miR5387b	cguggcuccucugguacu	19	*S. bicolor*
miR6225	bsy-miR6225-3p	aaaacgaacuggugcaggua	20	*S. bicolor*
miR7778	bsy-miR7778-3p	uugcgccggugucucccugcaac	23	*B. distachyon*
miR5050	bsy-miR5050	gugaacgaggaaaggcau	18	*T. aestivum*
miR5201	bsy-miR5201-3p	cgggcgaggccggcggcggcg	21	*B. distachyon*
miR820	bsy-miR820a	ucggccucgcgcucgcccuc	20	*O. sativa*
miR5181	bsy-miR5181-3p	cacuuauuauggaucagaggg	21	*A. tauschii*
miR1509	bsy-miR1509a-3p	uccggauuuuuuuucagauu	20	*M. truncatula*
miR159	bsy-miR159b-3p.3	uuugcaugaggaggacca	18	*B. distachyon*
miR169	bsy-miR169a-5p	aagccaaguugguucuggagu	21	*A. thaliana*
miR8634	bsy-miR8634	gugguauguggaagaugc	18	*G. raimondii*
miR1444	bsy-miR1444d	agaacguuuuaagaaguacauccag	25	*P. trichocarpa*
miR5181	bsy-miR5181-3p	cacuuauuauggaucagagguagu	24	*A. tauschii*
miR1222	bsy-miR1222a	uugaaggaaucacagagcgac	21	*P. patens*
miR5174	bsy-miR5174c-5p	ucccuccguuccaaauuaauug	22	*B. distachyon*
miR2927	bsy-miR2927	cgucgucggcgcagccgg	18	*O. sativa*
miR6207	bsy-miR6207	aggacgacgcuacuuccugcugc	23	*H. vulgare*

Table S2 Summary of novel miRNAs

miRNA location	Members	Sequence (5' to 3')
comp134939_c0_10133	novel-bsy-miR-1	ucugaagaugaaguauaucug
comp25586_c0_70228	novel-bsy-miR-2	aaaaaagauugagccgagu
comp19400_c0_33811	novel-bsy-miR-3	agugaguguaaaagcugacguggc
comp149885_c0_16793	novel-bsy-miR-4	agaauuauggaauggagggag
comp3470_c1_111252	novel-bsy-miR-5	ccuccgauccauaauaagugu
comp24509_c0_62522	novel-bsy-miR-6	ucgcuggagcucgcgugcaucccuu
comp73966_c0_129319	novel-bsy-miR-7	ugcggcugagaccggcgaau
comp21282_c0_44432	novel-bsy-miR-8	ccuccgaucggaaaagguugu
comp24294_c0_61116	novel-bsy-miR-9	ccucgcggaugcuggcgcg
comp22287_c0_49543	novel-bsy-miR-10	uagucgcugugggccaagccg
comp22287_c0_49542	novel-bsy-miR-11	uagucgcugugggccaagccg
comp149885_c0_16798	novel-bsy-miR-12	agaauuauggaacguagguag
comp417552_c0_117651	novel-bsy-miR-13	gugcgcgcggcgccgucgauc
comp20703_c0_42110	novel-bsy-miR-14	cuccgauuggaaaaaguuguc
comp9660_c0_144081	novel-bsy-miR-15	augguguauuacacaaacgug
comp230_c0_53881	novel-bsy-miR-16	acacuuauuauggaucggagg
comp46386_c0_120618	novel-bsy-miR-17	uuauccugacuuugguucacu
comp19417_c0_33904	novel-bsy-miR-18	ccucgucggcacggccgc
comp100249_c0_179	novel-bsy-miR-19	cuccgauccaaaauaaguguc
comp28138_c0_93266	novel-bsy-miR-20	acgugcggucccgguggugg
comp30236_c0_102344	novel-bsy-miR-21	uggacuucgccgacgucuucggcgg
comp17455_c0_28699	novel-bsy-miR-22	uuagcgauaauuaauauggga
comp25440_c0_68949	novel-bsy-miR-23	auagcauuugaggcacuuugg
comp31201_c0_104898	novel-bsy-miR-24	cgccauggaguggcuccaagaucca
comp28490_c0_94873	novel-bsy-miR-25	cacagccaccgcugccgccgcg
comp15354_c0_19166	novel-bsy-miR-26	ucgccuacuacugggagacgca
comp10274_c0_1790	novel-bsy-miR-27	uuuauaagacguuuuggcaag
comp26717_c0_80207	novel-bsy-miR-28	ucgccggccgccaccgagc
comp59396_c0_124670	novel-bsy-miR-29	ucgucuccgccgcgucgcucc
comp14214_c0_12079	novel-bsy-miR-30	aaaggcuuauauuguggaaca
comp15098_c0_17682	novel-bsy-miR-31	aucggagacugaugugcc
comp24365_c0_61690	novel-bsy-miR-32	uauccgugucaggcgugguggcagg
comp41330_c0_117446	novel-bsy-miR-33	augggcguaucauccuguauuaca
comp45964_c0_120432	novel-bsy-miR-34	ugcugggcugauccggggcc
comp27914_c0_91950	novel-bsy-miR-35	gccgcgcggcggggugcagggu
comp31072_c0_104552	novel-bsy-miR-36	cgcggacacggccaugguggcg
